# The SCHEIE Visual Field Grading System

**DOI:** 10.4172/2155-9570.1000651

**Published:** 2017-05-11

**Authors:** Prithvi S. Sankar, Laura O’Keefe, Daniel Choi, Rebecca Salowe, Eydie Miller-Ellis, Amanda Lehman, Victoria Addis, Meera Ramakrishnan, Vikas Natesh, Gideon Whitehead, Naira Khachatryan, Joan O’Brien

**Affiliations:** Scheie Eye Institute, University of Pennsylvania, Philadelphia, Pennsylvania, USA

**Keywords:** Glaucoma, Open-angle glaucoma, Visual fields, Visual field defects, Glaucomatous visual fields, Visual field grading, Standard automated perimetry

## Abstract

**Objective:**

No method of grading visual field (VF) defects has been widely accepted throughout the glaucoma community. The SCHEIE (Systematic Classification of Humphrey visual fields-Easy Interpretation and Evaluation) grading system for glaucomatous visual fields was created to convey qualitative and quantitative information regarding visual field defects in an objective, reproducible, and easily applicable manner for research purposes.

**Methods:**

The SCHEIE grading system is composed of a qualitative and quantitative score. The qualitative score consists of designation in one or more of the following categories: normal, central scotoma, paracentral scotoma, paracentral crescent, temporal quadrant, nasal quadrant, peripheral arcuate defect, expansive arcuate, or altitudinal defect. The quantitative component incorporates the Humphrey visual field index (VFI), location of visual defects for superior and inferior hemifields, and blind spot involvement. Accuracy and speed at grading using the qualitative and quantitative components was calculated for non-physician graders.

**Results:**

Graders had a median accuracy of 96.67% for their qualitative scores and a median accuracy of 98.75% for their quantitative scores. Graders took a mean of 56 seconds per visual field to assign a qualitative score and 20 seconds per visual field to assign a quantitative score.

**Conclusion:**

The SCHEIE grading system is a reproducible tool that combines qualitative and quantitative measurements to grade glaucomatous visual field defects. The system aims to standardize clinical staging and to make specific visual field defects more easily identifiable. Specific patterns of visual field loss may also be associated with genetic variants in future genetic analysis.

## Introduction

Glaucoma is the leading cause of irreversible blindness worldwide [1]. This disease is phenotypically heterogeneous, characterized by a spectrum of disease progression and severity. Monitoring disease progression and staging glaucoma patients is a crucial component of effective glaucoma management [2].

Glaucomatous damage can be quantified structurally and functionally. Structural glaucomatous damage is reflected by characteristic optic nerve and retinal nerve fiber layer change, measured using photography of the optic nerve and imaging techniques such as Confocal Scanning Laser Ophthalmoscopy, Scanning Laser Polarimetry, and Optical Coherence Tomography [3,4]. Functionally, glaucoma patients exhibit several characteristic patterns of visual field defects. The Humphrey Field Analyzer is the most widely accepted machine used to quantify visual field loss in clinical practice and research studies, with Standard Automated Perimetry (SAP) serving as the standard for assessing visual field damage [5]. However, there is no universally agreed upon standard for grading the diversity of patterns of visual field loss.

An objective method for classifying and grading visual field damage in glaucoma patients is needed. An optimal grading system should communicate sufficient information on defect characteristics, while being quick and easy to learn and apply. The system must also detect small changes over time, serving an accurate proxy of glaucoma progression [6]. Though numerous grading systems have been proposed, none fully meet these criteria and none have yet been widely accepted throughout the glaucoma community.

The Hodapp, Parrish, and Anderson System (HPA) system, a commonly used classification system, is based upon the overall extent of visual field depression (calculated by the mean deviation value), the number of defective points in the Humphrey Statpac-2 pattern deviation probability map of the 24-2, SITA-STANDARD test, and proximity to damage to fixation point [7]. In spite of its popularity, the HPA system has several limitations. There are only three stages of glaucoma classification: early, moderate, and severe. These broad categories make it difficult to monitor small, but meaningful, changes in functional loss over time [8,9]. The system does not provide any information regarding the location and depth of defects in the visual field [2]. Finally, the system is complicated and can be time-consuming, limiting its clinical use [2].

Mills et al. proposed a glaucoma staging system (GSS) that attempted to improve on the HPA system by adding 12 additional criteria and two additional stages [9]. While the new criteria made the GSS system more comprehensive, some of the criteria were found to be redundant and unnecessarily complicated in a subsequent study, thereby limiting its clinical utility [10].

A more continuous staging system was proposed by the Advanced Glaucoma Intervention Study (AGIS) [11]. The AGIS system is based on the number and depth of neighboring depressed test locations on the total deviation plot in the nasal area, upper hemifield, and lower hemifield [12]. Visual fields are scored between 0 and 20, with 0 indicating no measured defective points and 20 indicating severe visual field depression. AGIS visual fields are placed into five stages of severity based on the 20-point scale. However, as this system contains a high number of parameters, it is difficult to manually use in the clinic [6].

Other methods, such as Brusini’s Glaucoma Staging System, are based on SAP indices, such as the Visual Field (VF) indices [13]. VF indices are used to acquire information on both the severity of defects and the type of damage. However, this method does not supply information regarding the location, shape, and morphology of defects, leading to overlapping classifications [2]. The Enhanced Glaucoma Staging System was derived from the GSS and is considered to be more easily implemented with more severe staging of abnormal fields [12,14].

The University of São Paulo Glaucoma Visual Field Staging System (USP-GVFSS), proposed in 2009, uses four main parameters. These parameters include the Visual Field Index (VFI-a Humphrey Visual Field Test value), location of the defect, involvement of the blind spot, and number of hemifields affected [2]. Limitations include the lack of information about which hemifield (superior or inferior) has visual field loss, difficulty with separate monitoring of the superior and inferior hemifields, and absence of a qualitative component describing patterns of visual field loss. While easy to quickly apply in the clinic, this system has little utility as a research tool.

The SCHEIE (Systematic Classification of Humphrey visual fields-Easy Interpretation and Evaluation) grading system improves on the quantitative aspects of the USP-GVFSS and combines them with qualitative measurements for grading glaucomatous visual field defects. Our aim was to standardize visual field grading, to make specific visual field defects more easily identifiable, and to correlate genetic variants with visual fields in a large glaucoma population.

## Methods

### Format of grading system

The SCHEIE grading system is composed of a qualitative and quantitative score, described in the sections below (see video at: https://www.omicsonline.org/open-access/vedio/the-scheie-visual-field-grading-system.mp4). Individual points on a pattern deviation plot are deemed significant based on their probability of being normal (Table 1A). A point with <5% probability is not considered significant [15]. A point with <2% probability is only considered significant if adjacent to a <2%, <1% and/or <0.5% point in any horizontal, vertical, or diagonal direction. Points with <1% and <0.5% probability are considered significant by themselves.

### Qualitative grading

All visual fields are initially categorized based on a qualitative description of the pattern of visual field depression. The superior and inferior hemifields are considered separately. The nine potential categories are described below.

#### Normal

**Figure 1:**
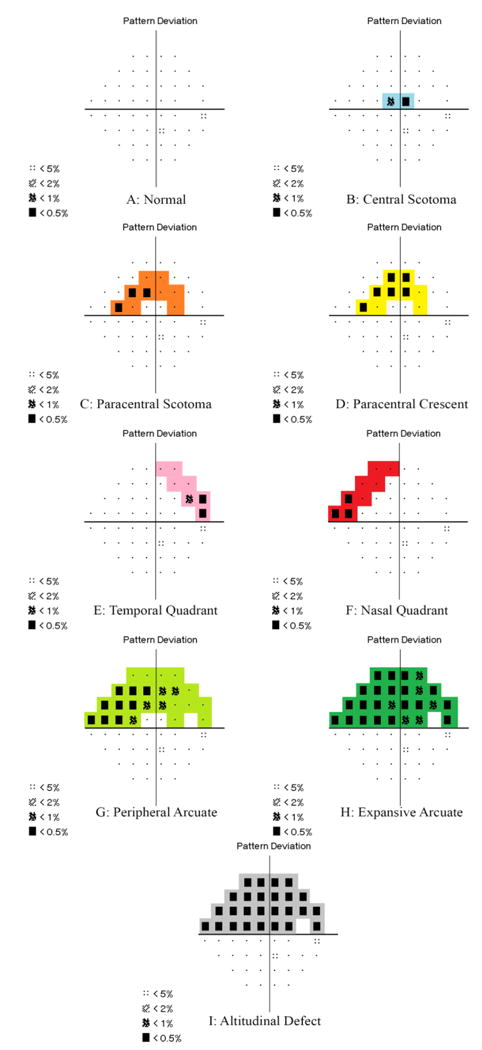
Nine Categories for Qualitative Grade: Normal (A), Central Scotoma (B), Paracentral Scotoma (C), Paracentral Crescent (D), Temporal Quadrant (E), Nasal Quadrant (F), Peripheral Arcuate (G), Expansive Arcuate (H), Altitudinal Defect (I). Note: Defects are only shown in the superior hemifield.

No defects anywhere on the field (Figure 1A).

#### Central scotoma

Defect within the central 5 degrees (Figure 1B). Note: If an adjoining Paracentral Scotoma or Paracentral Crescent is present, the entire defect is considered a Central Scotoma.

#### Paracentral scotoma

Defects within the paracentral region (points outside the central 5 degrees and within 10 degrees) that do not cross the midline (Figure 1C).

#### Paracentral crescent

Defects in the paracentral region that do cross the midline (Figure 1D).

#### Temporal quadrant

Any defect within the temporal zone (Figure 1E). Note: If a paracentral scotoma is adjoining, the defect is considered under the umbrella of temporal quadrant. If a paracentral crescent is present, the defect is considered either a peripheral or expansive arcuate defect.

#### Nasal quadrant

Any defect within the nasal zone (Figure 1F). Note: If a paracentral scotoma is adjoining, the defect is considered under the umbrella of nasal quadrant. If a paracentral crescent is present, the defect is considered a peripheral or expansive arcuate defect.

#### Peripheral arcuate

A combination of defects that must cross the midline and does not include the central two points (Figure 1G). Note: This defect may combine temporal and nasal quadrants that meet at the meridian or a paracentral crescent plus a temporal or nasal quadrant.

#### Expansive arcuate

A combination of defects that must cross the midline, and includes adjoining defects in the central scotoma region, paracentral region, and temporal or nasal quadrant region. At least one point must be <1% or greater (Figure 1H).

#### Altitudinal defect

All of the points above or below the horizontal meridian have a defect of <0.5% (Figure 1I).

Defects may fit the criteria for more than one type of defect as they worsen. If the defects are not adjoining, they may be listed concurrently (i.e. a temporal quadrant and a nasal quadrant). If the defects are adjoining, they may be combined into a broader category (i.e. paracentral crescent adjacent to a nasal quadrant is considered a peripheral arcuate defect). It is possible to have two distinct, non-adjacent defects in the same category (Eg: temporal quadrant × 2).

### Quantitative grading

Once the visual fields have been qualitatively assessed, they should be grouped together based on their category. Visual fields within each group are then given a numeric grade, which incorporates the Humphrey visual field index (VFI), location of visual defects for superior and inferior hemifields, and blind spot involvement:

$$\text{Grade}\;\text{Template}\;=\text{VFI}\;\text{Score}/\frac{\text{Superior}\;\text{Hemifield}}{\text{Inferior}\;\text{Hemifield}}/\text{Blind}\;\text{Spot}\;\text{Involvement}$$

The individual components of this score are detailed below:

#### VFI score

VFI is a parameter calculated by the Humphrey Field Analyzer that indicates the amount of visual field loss present in a given patient. A measure of severity, VFI is a continuous variable given as a percentage ranging from 100% (normal) to 0% (parametrically blind) [16].

#### Hemifield involvement

**Figure 2:**
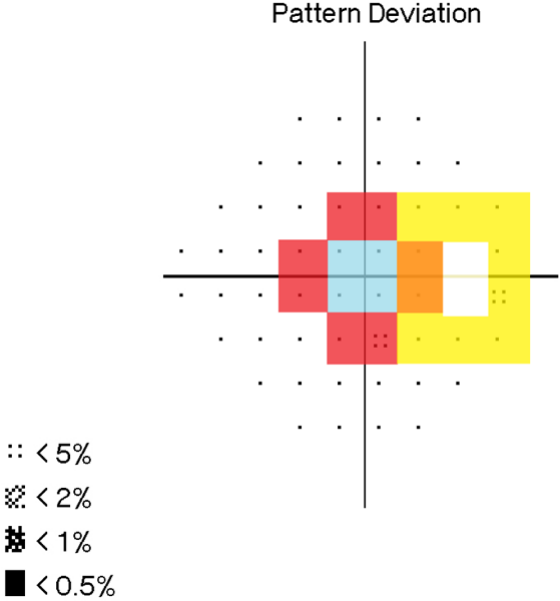
Illustration of Points within 5° (blue), within 10° (red/orange), and Adjacent to Blind Spot (yellow/orange).

The hemifield portion of the grade is determined by examining the location of significantly depressed points on the pattern standard deviation probability map. Points count as significant based on their probability, detailed in the criteria in Table 1A. Each hemifield is given a score of 5, 10, 30, or NL based on the most central location of significant points in the field (Table 1B). In Figure 2, the blue square contains the central 5 degrees; the red/orange cross represents the outer limit for defects in the central 10 degrees; and the remainder of the field comprises the outer 30 degrees. A hemifield is marked NL if there are no significant points.

#### Blind spot involvement

If a significant depressed point is in contact with the blind spot, a plus sign (+) is assigned. Otherwise, a minus sign (−) is assigned. The points highlighted in yellow and orange in Figure 2 are considered to be adjacent to the blind spot.

### Examples of grading system

Four examples of graded visual fields are described below.

**Figure 3:**
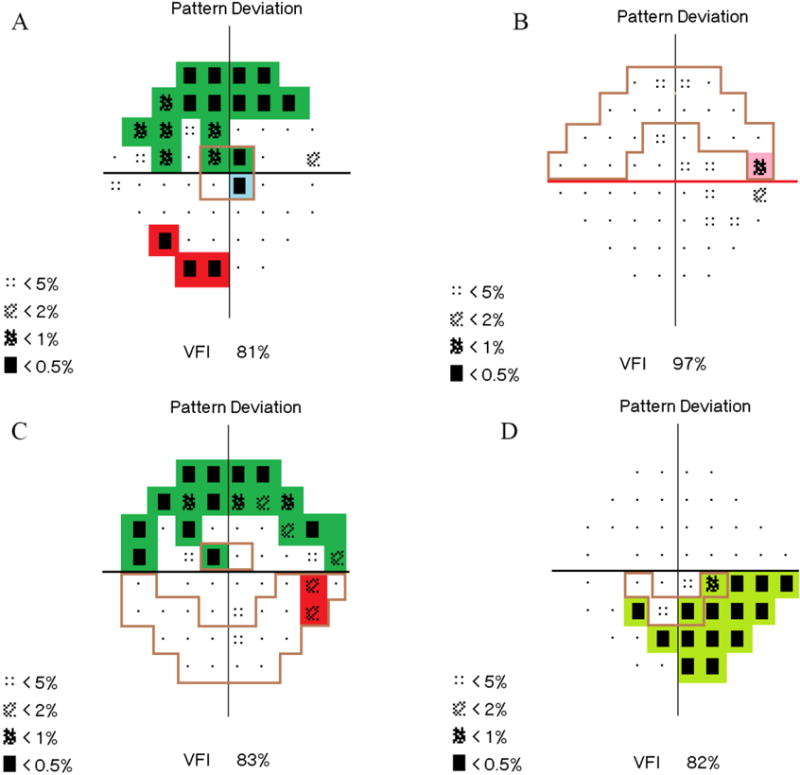
Four Examples of Grading System (A, B, C, D). The defects are highlighted (qualitative grade) and outlined (hemifield portion of quantitative grade).

***Example 1*** (Figure 3A)

#### Qualitative grade

##### Superior

Adjoining defects are present in the central, paracentral, nasal quadrant, and temporal quadrant regions and cross the midline. The grade is expansive arcuate.

##### Inferior

A<0.5% point is present in a center point. Three adjoining defects are also present in the nasal quadrant region. The grade is central scotoma and nasal quadrant.

##### Final qualitative grade

Superior expansive arcuate/inferior central scotoma and nasal quadrant.

#### Quantitative grade

##### VFI

81%

##### Superior

A<0.5% point is located within 5 degrees. The superior hemifield grade is 5.

##### Inferior

A<0.5% point is located within 5 degrees. The inferior hemifield grade is 5.

##### Blind spot involvement

The only point adjacent to the blind spot is an isolated <2% point. A <2% point is not considered significant on its own; it needs a <2%, <1%, or <0.5% point adjacent to it in the same hemifield to be activated. Therefore, there is no blind spot involvement (−).

##### Final quantitative grade

81/5/5/−

##### SCHEIE grade

Superior expansive arcuate/inferior central scotoma and nasal quadrant: 81/ 5/5/−

***Example 2* (**Figure 3B).

#### Qualitative grade

##### Superior

There is a <1% point in the temporal quadrant region. The grade is temporal quadrant.

##### Inferior

A <2% point occurs in the inferior hemifield, with no <2%, <1%, or <0.5% point adjacent in the same hemifield to activate it. (It is not activated by the <1% point, which is part of the superior hemifield). The grade is normal.

##### Final qualitative grade

Superior temporal quadrant/inferior normal.

#### Quantitative grade

##### VFI

97%

##### Superior

A<1% point occurs within 30 degrees. There is no significant point more central than this point, so the grade is 30.

##### Inferior

A<2% point occurs in the inferior hemifield, with no <2%, <1%, or <0.5% point adjacent in the same hemifield to activate it. (It is not activated by the <1% point, which is part of the superior hemifield). The grade is NL.

##### Blind spot involvement

The<1% point is significant and adjacent to the blind spot. Therefore, there is positive blind spot involvement (+).

##### Final quantitative grade

97/30/NL/+

##### SCHEIE grade

Superior temporal quadrant/inferior normal: 97/30/NL/+

***Example 3* (**Figure 3C)

#### Qualitative grade

##### Superior

Adjoining defects are present in the central, paracentral, nasal quadrant, and temporal quadrant and cross the midline. The grade is expansive arcuate.

##### Inferior

There are two adjacent <2% points in the nasal quadrant region. The grade is nasal quadrant.

##### Final qualitative grade

Superior expansive arcuate/inferior nasal quadrant.

#### Quantitative grade

##### VFI

83%

##### Superior

A<0.5% point occurs within 5 degrees. The grade is 5.

##### Inferior

There is a<5% point within the central 10 degrees inferiorly, which is not considered significant. There are two adjacent<2% points in the outer 30 degrees, which activate each other. The grade is 30.

##### Blind spot involvement

There are multiple <0.5% points adjacent to the blind spot. Therefore, there is positive blind spot involvement (+).

##### Final quantitative grade

83/5/30/+

##### SCHEIE grade

Superior expansive arcuate/inferior nasal quadrant: 83/5/30 /+

***Example 4* (**Figure 3D)

#### Qualitative grade

##### Superior

There are no significant points in the superior hemifield. The grade is normal.

##### Inferior

There is an adjoining defect that crosses the midline and does not include the central 5 degree points. The grade is peripheral arcuate.

##### Final qualitative grade

Superior normal/inferior peripheral arcuate.

#### Quantitative grade

##### VFI

82%

##### Superior

There are no significant points in the superior hemifield. The grade is NL.

##### Inferior

A<0.5% point occurs within 10 degrees. The grade is 10.

##### Blind spot involvement

A<0.5% point adjacent to the blind spot. Therefore, there is positive blind spot (+).

##### Final quantitative grade

82/ NL/10 /+

##### SCHEIE grade

Superior normal/inferior peripheral arcuate: 82/ NL/10/+

### Assessment of accuracy

To assess the accuracy of the SCHEIE system, graders were trained by a glaucoma specialist on how to use the system. Graders were college graduates without a medical background who were not familiar with visual field analysis reports or this specific grading system. After approximately 40 minutes of training, which included discussion of visual field examples, each masked grader independently graded visual fields.

For the qualitative portion, six trained graders each graded 30 visual fields. Each grader’s responses were compared to the master key created by a glaucoma specialist to assess accuracy. Accuracy rates were calculated using the following formula: #of correctly graded fields/30. All components of the final grade needed to be correct for the answer to be counted as correct.

For the quantitative portion, ten trained graders each graded 50 visual fields (distinct fields from qualitative portion). Accuracy rates were calculated for each component of the composite quantitative score using the following formula: #of correctly graded (insert individual component)/50, with components including: VFI (a number 1–100), superior hemifield (5, 10, 30, NL), inferior hemifield (5, 10, 30, NL), and blind spot involvement score (+ or −). Accuracy rates were also calculated for the overall score using the formula: total number correct/50. All four components needed to be correct for the overall score to be counted as correct. All graders were timed.

## Results

Graders had a median accuracy of 96.67% for their overall qualitative scores on 30 visual fields and a median accuracy of 98.33% on the 60 individual hemifields (Table 2A). Graders took a mean of 27.88 minutes for qualitative grading (average of 56 seconds per visual field). Graders had a median accuracy of 98.75% for their overall quantitative scores on 50 visual fields (Table 2B). When examining the breakdown of the overall quantitative score, graders had a median accuracy of 100.00% on VFI, 98.00% on superior hemifield, 98.00% on inferior hemifield, and 98.00% on blind spot involvement. Graders took a mean of 16.55 minutes to grade the 50 visual fields (average of 20 seconds per visual field).

## Discussion

The SCHEIE grading system is a novel method of scoring glaucomatous visual fields and improves on existing grading systems in several key ways. Firstly, the inclusion of qualitative categories as part of the score allows patients with similar patterns of visual field loss to be grouped together. Stratifying patients by qualitative defects avoids some of the clinical pitfalls of a solely quantitative score, produced by systems such as the USF-GVFSS. A system based on numerically grading loss according to the degree of central field depression will generally assign a worse score to a central scotoma than an arcuate pattern of glaucomatous damage. Thus, if one patient has a minor central scotoma while another has a dense arcuate defect, the patient with the damaged arcuate defect will have a seemingly better “score,” which does not correlate with the actual clinical comparison. Including a qualitative component in the SCHEIE score will help to avoid these limitations.

Secondly, the quantitative component of the SCHEIE grading system may be an effective tool for longitudinally monitoring the progression of glaucoma. Any meaningful changes in function, often manifested by more extensive central visual field involvement in a patient, can be assessed by a change in the hemifield scores. Furthermore, the SCHEIE grading system provides information on the hemifield location of defects, unlike several existing systems, which allows for separate monitoring of the superior and inferior hemifields. Further research is needed to evaluate the application of this grading system to the analysis of glaucoma progression.

Finally, our study showed that the SCHEIE grading system is easy to learn and apply, as demonstrated by the short session needed to train graders and complete grading. The high accuracy of non-physician graders also shows that the system is reproducible and accurate among trained graders, without need for an ophthalmology background. This lends itself to use in research studies, where large-scale grading and categorization of visual fields is required. In the future, the SCHEIE grading system could also lend itself to automated grading.

The SCHEIE grading system has important ramifications for future glaucoma genetic studies. The SCHEIE grading system makes the visual field a quantitative trait, which can then be associated with large numbers of genetic variants that correlate with the development or progression of glaucoma. Qualitative groupings of patients can also be investigated to see which genes are implicated in different patterns of glaucomatous damage. This information may lead to important therapeutic developments that offer a more tailored approach to treating glaucoma patients based on their specific phenotype. Existing systems, such as the HPA system, often have few broad categories that are difficult to use for this function.

Limitations of the SCHEIE grading system include that it is only compatible with Humphrey Visual Fields (not Octopus, Goldmann, or other types of field tests), as VFI and the points used to score the visual fields are particular to the Humphrey system. VFI is also not as sensitive for early disease [16]. In the future, a modified or equivalent classification that uses parameters unique to another perimeter could be incorporated into the system. The SCHEIE system also depends on the reliability of the test. Finally, the SCHEIE visual field system was developed to objectively analyze visual fields in the research setting. There is a learning curve to applying any new system, including this one. Although we believe that this system lends itself to clinical applications as well, it has not yet been broadly applied.

## Supplementary Material

Suppl Video

## Figures and Tables

**Table 1:** SCHEIE grading system definitions, including **A)** definition of significant points, and **B)** determination of hemifield grade based on location of significant points.

A: Definition of Significant Points	
**Point Value**	**Rule**
<0.5%	One point counts.
<1%	One point counts.
<2%	A point only counts if it is next to a <2%, <1%, or <0.5% point in the same hemifield. The points may be in contact diagonally.
<5%	This point never counts for itself and never causes a <2% point to count.
**B: Determination of Hemifield Grade**	
**Grade**	**Definition**
Normal (NL)	No depressed points anywhere on the field.
30	Depressed points occur, but not within 10 degrees of fixation.
10	Depressed points occur within 10 degrees, but not 5 degrees of fixation.
5	Depressed points occur within 5 degrees of fixation.

**Table 2:** Accuracy of Scheie Grading System, including **A)** Accuracy of 6 non-physician graders on 30 visual fields for qualitative component, and **B)** Accuracy of 10 non-physician graders on 50 visual fields for quantitative component.

Graders	# 1	# 2	# 3	# 4	# 5	# 6	# 7	# 8	# 9	# 10	Mean	Median
**A: Qualitative Component**												
Full fields	93.33%	100.00%	86.67%	96.67%	100.00%	100.00%	N/A	N/A	N/A	N/A	96.11%	96.67%
Ind. hemifields	96.67%	100.00%	93.33%	98.33%	100.00%	100.00%	N/A	N/A	N/A	N/A	98.06%	98.33%
**B: Quantitative Component**												
VFI	100.00%	100.00%	100.00%	100.00%	98.00%	100.00%	100.00%	100.00%	100.00%	100.00%	99.80%	100.00%
SUP	96.00%	100.00%	100.00%	100.00%	94.00%	98.00%	98.00%	98.00%	100.00%	94.00%	97.80%	98.00%
INF	98.00%	96.00%	96.00%	98.00%	92.00%	100.00%	100.00%	100.00%	98.00%	96.00%	97.40%	98.00%
Blind Spot	100.00%	98.00%	98.00%	98.00%	76.00%	100.00%	100.00%	98.00%	98.00%	96.00%	96.20%	98.00%
Total	98.50%	98.50%	98.50%	99.00%	90.00%	99.50%	99.50%	99.00%	99.00%	96.50%	97.80%	98.75%

## OMICS International: Open Access Publication Benefits & Features

### Unique features

- Increased global visibility of articles through worldwide distribution and indexing
- Showcasing recent research output in a timely and updated manner
- Special issues on the current trends of scientific research

### Special features

- 700+ Open Access Journals
- 50,000+ editorial team
- Rapid review process
- Quality and quick editorial, review and publication processing
- Indexing at major indexing services
- Sharing Option: Social Networking Enabled
- Authors, Reviewers and Editors rewarded with online Scientific Credits
- Better discount for your subsequent articles

Submit your manuscript at: http://www.omicsonline.org/submission/
